# Investigating the contribution of task and response repetitions to the sequential modulations of attentional cueing effects

**DOI:** 10.1007/s00426-017-0950-y

**Published:** 2017-11-29

**Authors:** Ulrich Ansorge, Davood G. Gozli, Florian Goller

**Affiliations:** 1 Faculty of Psychology, University of Vienna, Liebiggasse 5, 1010 Vienna, Austria; 2 Department of Psychology, University of Macau, Macau S.A.R., China

## Abstract

We tested the nature of validity sequence effects. During visual search for targets, target-preceding peripheral cues at target position (valid condition) facilitate search relative to cues away from the target (invalid condition). This validity effect (i.e., advantage in valid compared to invalid conditions) is observed for cues that are not predictive of the target, and it reflects the cue’s capture of attention. Importantly, the validity effect is stronger following valid than invalid trials. The underlying causes of this validity sequence effect are unknown. We, therefore, tested if the validity sequence effect reflected trial-to-trial priming or event-file coding. According to these explanations, full trial-to-trial repetitions and full changes of all stimulus features or of all stimulus and response features, respectively, would account for the validity sequence effect. However, the validity sequence effect could also reflect the participants’ retention of a recently helpful cue (i.e., after a valid trial) and/or their suppression of a recently harmful cue (i.e., after an invalid trial). Here, to contrastively test these theories, from trial to trial, the tasks are repeated or switched. The results demonstrated that, under certain conditions, the validity sequence effect can survive task-switching (Experiments 1 and 2), which supports the retention/suppression account. When the tasks were strongly distinguished, however, the validity sequence effect did not survive task-switching (Experiment 3), which supports the event-coding account. Together, the results suggest that task structure can determine the impact of cue processing on subsequent trials, and the extent to which it reflects event-file coding.

## Introduction

One robust finding across cognitive processes and tasks concerns the influence of cognitive conflict history on conflict-elicited effects (Botvinick, Carter, Braver, Barch, & Cohen, 2001; Egner, 2008; Gratton, Coles, & Donchin, 1992; for a review, see Duthoo, Abrahamse, Braem, Boehler, & Notebaert, 2014): conflict in the most recent trial *n*-1 diminishes conflict effects in the current trial *n*. Take the flanker task, where participants respond to a target and (try to) ignore adjacent distractors. Here, participants respond faster with congruent distractors indicating the target response than with incongruent distractors indicating a different response. This congruence effect (incongruent reaction time [RT] minus congruent RT) reflects conflict between response activation by targets and incongruent distractors (Gratton, Coles, Sirevaag, Eriksen, & Donchin, 1998). Important in the present context, there is a congruence sequence effect: congruence effects are stronger following congruent than incongruent trials (Gratton et al., 1992).

The current study is concerned with the possible origins of one variant of the conflict sequence effect: the sequential modulation of the attentional cueing (or validity) effect, henceforth, referred to as validity sequence effect. When participants have to search for a target presented unforeseeably at one out of several positions, in the valid condition, presenting a cue prior to the target and at target position facilitates responding to this target. This facilitation is found relative to an invalid condition, in which cue and target are presented at alternative positions. With peripheral cues, such validity effects are found with short cue-target intervals even if the cue is not predictive of the most likely target position. This validity effect has been attributed to the orienting of attention to the cue, such that target search is facilitated in valid conditions where attention would be oriented toward the target too, compared to invalid conditions where attention to the cue would be directed away from the target (Posner, 1980). The same effect can also be found with central cues, such as arrows pointing towards one of the potential target positions, at least if central cues are predictive of the most likely target position (Müller & Rabbitt, 1989). Most importantly, a handful of studies suggested validity sequence effects, with stronger validity effects following *n*-1 valid than following *n*-1 invalid trials (Goller & Ansorge, 2015; Jongen & Smulders, 2007; Mordkoff, Halterman, & Chen, 2008; Qian, Shinomori, & Song, 2012; see also Müller, Geyer, Zehetleitner, & Krummenacher, 2009), but the exact origin of this sequential modulation is not yet understood.

Here, we wanted to close this gap in the literature, and started to test some of the possible explanations of the validity sequence effect. For our test, we considered several of the explanations that have been given for conflict sequence effects in other tasks than the cueing task, such as more inhibition of irrelevant information (here: the cues) following conflicting than following non-conflicting trials (Botvinick et al., 2001), more expectation of helpful information by the accessory stimuli or features following non-conflicting than conflicting trials (Gratton et al., 1992), better learning of relevant target features in incongruent trials (Verguts & Notebaert, 2008), less priming of target features in incongruent and more priming of target features in congruent trials *n* (Mayr, Awh, & Laurey, 2003), or more processing costs associated with the representations of targets and responses in joint event-files in incongruent trials following a congruent trial *n*-1, and in congruent trials following an incongruent trial *n*-1, than in some of the congruent–congruent and incongruent–incongruent *n*-1-to-*n* sequences (Hommel, Proctor, & Vu, 2004; Schumacher & Hazeltine, 2016).

Of importance for the current study, only two of these theories argue for a role of trial-to-trial feature repetitions in the sequence effects: the priming account and the event-file coding account. According to the priming account, processing of a feature in trial *n* is expedited if the same feature was used in trial *n*-1. Such processing advantages by priming would be the largest in trials with full trial-to-trial repetitions of all relevant and irrelevant stimulus features—that is, some of the congruent-to-congruent (here: valid-to-valid) and some of the incongruent-to-incongruent (here: invalid-to-invalid) trial-to-trial sequences. By definition, congruent-to-incongruent and incongruent-to-congruent sequences always contain at least one feature change from trial to trial. Critically, because conflict effects (here: cueing or validity effects) in trial *n* are calculated as incongruent minus congruent performance (here: invalid minus valid performance), trial-to-trial priming benefits would modulate the cueing or validity effect in trial *n*: priming would increase the *n*-validity effect where it brings the *n*-valid response times (RTs) down—that is, following *n*-1 valid trials; and priming would decrease the *n*-validity effect where it brings the *n*-invalid RTs down—that is, following *n*-1 invalid trials.

To test the influence of priming, in the first two experiments, we used different tasks with differently colored targets and tested if the validity sequence effect is restricted to task-repetition conditions, which should be the case if the priming explanation holds true. Because target colors would only be primed when the task repeated from trial *n*-1 to *n*, finding a validity sequence effect following trial-to-trial changes of the task would be at variance with the priming explanation.

Related but not identical to the priming account is the event-file coding explanation, according to which participants code all stimulus and response features in a trial into one joint-event file representing the trial. The key difference between the priming and the event-file coding explanations is their assumptions about the units of representation underlying task performance and, by implication, the nature of priming. Whereas the former takes the units to be separate stimulus and response features, each susceptible to independent priming, the latter takes combination of stimulus–response features (event files) as the units of representation. From trial *n*-1 to *n*, repetition of a feature that belongs to an event file incurs an advantage only if other features belonging to the event file also repeat, whereas a recombination cost incurs if only part of the features from trial *n*-1 is re-used to code the event in trial *n* (Hommel et al., 2004). Importantly, recombination costs would be lowest in full trial-to-trial event repetitions and full trial-to trial event changes. In terms of cue validity, full trial-to-trial repetitions are restricted to congruent-to-congruent (here: valid-to-valid) and incongruent-to-incongruent (here: invalid-to-invalid) sequences. Thus, as in the case of priming, low recombination costs in a valid trial *n* would increase the *n*-validity effect following an *n*-1 valid trial, but low costs in an invalid trial *n* would decrease the *n*-validity effect following an *n*-1 invalid trial only in trial-to-trial full repetition conditions. That is, a validity sequence effect without trial-to-trial full repetitions would be at variance with an event-file coding explanation.

Research has demonstrated that, at least with response-conflicting stimuli, conflict sequence effects are not entirely explained by inter-trial priming or event-file coding; the congruence sequence effect has been even found without trial-to-trial repetitions of target features (e.g., of positions, see Wühr, 2005) or of responses (e.g., Kunde & Wühr, 2006). This, however, has not been tested for validity sequence effects. In addition, recently, the event-file coding theory has been extended to include task-set dependent flexibility: the task-file coding explanation makes ultimately very similar predictions as the event-file coding account, but it emphasizes that sequential modulations by event-file coding can depend on the participants’ representation of two successive trials as belonging to the same task (Schumacher & Hazeltine, 2016). In many cases, stimuli, responses, or stimulus–response (SR) mappings in an experiment only differ partly and to varying degrees from one another, allowing their representations by the participants in one joint task set or in alternative task sets. This means that humans show flexibility in how they represent different SR rules. Instructions not only play a role for how tasks are represented, as they emphasize differences or commonalities across different SR rules, but also if or if not salient or advance information allows participants to reduce their uncertainty about what exact SR rules apply next. Suppose, in a 4-alternative forced-choice (4-AFC) task, we begin each trial by presenting the participant with one of two easily distinguishable features (e.g., the colors red and blue), enabling her to simplify the upcoming SR conditions from a 4-AFC to a 2-AFC condition. Very likely, the task-identifying features would then be integrated into two different task sets, each associated with a relatively simpler SR mapping. A demonstration was provided by Hazeltine et al. (2011), who used a task in which stimulus modality (i.e., auditory or visual) was repeated or switched from trial to trial. When stimulus modalities were uniquely associated with different SR rules (or tasks), they found a conflict sequence effect that was restricted to trial-to-trial repetitions of stimulus modalities. By contrast, when both modalities were not as predictive of the SR rule and, thus, when both modalities were represented within a joint task set, the conflict sequence effect persisted through the trial-to-trial modality changes (Hazeltine, Lightman, Schwarb, & Schumacher, 2011, Experiment 4). This is in line with the extension of event-file coding to tasks.

To test the influence of event coding, all analyses of the present study were made with the additional variable trial-to-trial response switching (response switches vs. response repetitions) to investigate if the validity sequence effect is restricted to response- and task-repetition conditions. This should be the case, if the event-file coding explanation holds true because full repetitions of the events would only be possible if the responses would also repeat from *n*-1 to *n*. In contrast, both the general suppression of irrelevant information following an *n*-1 incongruent trial and the expectation of helpful information following an *n*-1 congruent trial would allow for conflict sequence effects that are independent of trial-to-trial stimulus and/or response repetitions (e.g., Freitas, Bahar, Yang, & Banai, 2007).

To further test the task-coding principle, between experiments, we varied the strength of overall association between cues and different SR rules. That is, different cues would sometimes predict different currently pertaining SR rules (Experiment 3). This should invite representation of the different cues in different task sets. Alternatively, a similar cue could occur with different current SR rules. This should not invite representation of the cue in different task sets. Whereas, the same cue was used for different SR rules in Experiments 1 and 2, different cues were used for different SR rules in Experiment 3. If the task-coding principle holds true, we might find more evidence for event-file coding principles in experiments with different cues for different SR rules than in experiments with similar cues for different SR rules (cf. Hazeltine et al., 2011). In contrast, again, a general inhibition of irrelevant information following *n*-1 incongruent trials or a general expectation of helpful accessory information following *n*-1 congruent trials could work independently of the particular task sets employed in two successive trials and, thus, might not be affected by the degree to which cues are predictive of SR rules.

## Experiment 1

### Methods

#### Participants

Thirty-six participants (27 female, 9 male, aged 18 to 32 years; *M*_Age_ = 22.1 years) participated. Informed consent was obtained. All participants had normal or corrected-to-normal vision and intact color vision.

#### Stimuli and procedure

Stimuli were presented on a 15-inch, color VGA monitor of 59.1 Hz refresh rate. Participants sat 57 cm from the screen in a dimly lit room, with their heads supported by a chin rest. RTs and response identities were registered via keys #4 and #6 of the numeric key pad of a standard QWERTZ computer keyboard in front of the participants. All keys were operated with the right index finger.

**Fig. 1:**
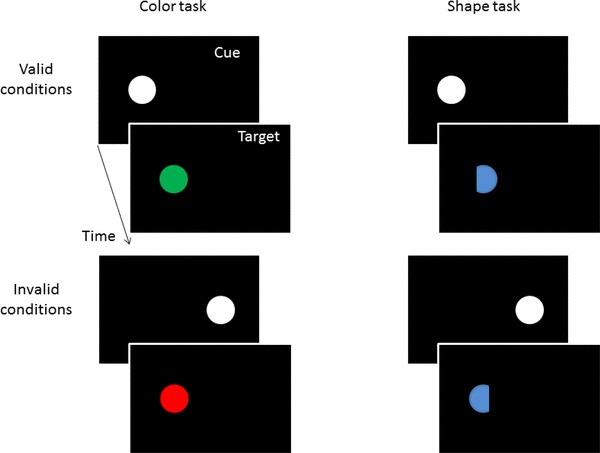
Schematic examples of the sequence of events in valid and invalid trials of the color task (on the left) and of the shape task (on the right) in Experiment 1. Stimuli are not drawn to scale

On each trial, unforeseeably but equally likely for the participants one of two tasks was administered: a color or a shape task. All trials (see Fig. 1) began by the participant pressing the central key #5 on the number pad. This prompted the concomitant presentation of a fixation cross at screen center and a 1° white (48 cd/m^2^) diameter disk as a peripheral cue at 4.0° left or right of fixation for 50 ms. After cue offset, the fixation cross was presented for 100 ms, and with a cue-target onset asynchrony of 150 ms a target disk (1.0°) was shown. The target was equally likely left and right, and equally likely at the cued position (valid condition) or on the opposite side (invalid condition). In the color task, the target (~ 26 cd/m^2^) was either a red (CIE color coordinates 0.64/0.35) or a green (0.26/0.56) disk. Half the participants pressed right (#6) for red and left (#4) for green targets, and this mapping was reversed for the other half of the participants. In the shape task, targets were blue (0.15/0.11) and had their left or right segment missing. These blue targets had the same luminance as the other color stimuli. Participants discriminated between these two target shapes by pressing the right button for targets missing their right and the left button for targets missing their left.

In both tasks, targets were shown for 190 ms and participants had 1.5 s for their responses. Feedback (750 ms) was provided if the response was too slow (> 1.250 ms, “Respond faster!”) or if an error was made (“Wrong answer!”).

By orthogonally crossing target identities, target positions, and their respective valid and invalid cue conditions, we studied *n*-validity (*n*-valid vs. *n*-invalid), *n*-1 validity (*n*-1 valid vs. *n*-1 invalid), *n*-task (color task vs. shape task) *n*-1-to-*n* task repetition (task repetition vs. switch), and *n*-1-to-*n* response repetition (response repetition vs. switch). There were 12 blocks of 96 trials, with 24 repetitions of the combinations of two tasks (color task; shape task) × two cueing conditions (valid; invalid). Together with verbal instructions and practice at the start, the experiment took 60 to 75 min.

### Results

#### Reaction times

Trials following an error in *n*-1 (4.7%) or with RTs below 100 ms or above 1 s (another 4.9%) were excluded. We conducted a repeated-measurements analysis of variance (ANOVA) of mean correct RTs, with variables (1) *n*-validity (*n*-valid; *n*-invalid), (2) *n*-1 validity (*n*-1 valid; *n*-1 invalid), (3) task (color task; shape task), (4) trial-to-trial task repetition (task repetition; task switch), (5) and trial-to-trial response repetition (response repetition; response switch).

**Fig. 2:**
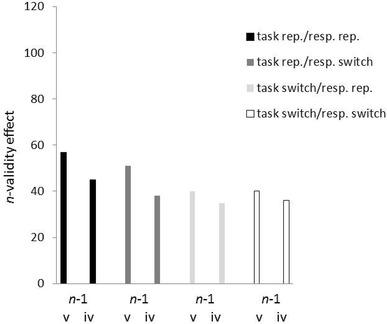
*n*-validity effects (*n*-invalid reaction time [RT] minus *n*-valid RT; in ms) on the abscissa as a function of *n*-1 validity (*n*-1 valid vs. *n*-1 invalid) on the ordinate, and of trial-to-trial task repetition (repetition: black and dark gray bars vs. switch: light gray and white bars) and response repetition (repetition: black and light gray bars vs. switch: dark gray and white bars). Data from Experiment 1. *v* valid, *i* invalid, *rep*. repetition, *swi*. switch

The ANOVA of the correct RTs led to a significant main effect of *n*-validity, *F*(1, 35) = 86.06, *p* < 0.01, partial *η*^2^ = 0.71, and to a significant *n*-validity × *n*-1 validity interaction, *F*(1, 35) = 15.71, *p* < 0.01, partial *η*^2^ = 0.31. RTs were lower under valid (RT = 601 ms) than under invalid (RT = 643 ms) conditions, and this validity effect was stronger following *n*-1 valid (validity effect = 47 ms, *t*[35] = 9.44, *p* < 0.01, for the test against zero) than *n*-1 invalid (validity effect = 38 ms, *t*[35] = 8.64, *p* < 0.01, for the test against zero) trials; *t*(35) = 3.96, *p* < 0.01, for the difference between the validity effects. Numerically, there was a tendency for lower validity sequence effects in task-switching conditions (see Fig. 2), but the two-way interaction of *n*-validity and *n*-1 validity was not involved in any higher-order interactions with task repetition and response repetition, all nonsignificant interactions *F*s < 3.67, all *p*s > 0.06.

*n*-validity also interacted significantly with task repetition, *F*(1, 35) = 28.20, *p* < 0.01, partial *η*^2^ = 0.45, and in a three-way interaction with task repetition and response repetition, *F*(1, 35) = 5.56, *p* < 0.05, partial *η*^2^ = 0.14. The *n*-validity effect was stronger when the task repeated (validity effect = 48 ms, *t*[35] = 9.22, *p* < 0.01, for the test against zero) than when it switched (validity effect = 37 ms, *t*[35] = 8.97, *p* < 0.01, for the test against zero); *t*(35) = 5.31, *p* < 0.01, for the difference between the validity effects. It was also numerically stronger when the task *and* the response repeated (validity effect = 51 ms, *t*[35] = 8.49, *p* < 0.01, for the test against zero) than when the task switched and the response was repeated (validity effect = 36 ms, *t*[35] = 8.27, *p* < 0.01, for the test against zero), when the response switched and the task repeated (validity effect = 45 ms, *t*[35] = 9.21, *p* < 0.01, for the test against zero), or when both task and response switched (validity effect = 39 ms, *t*[35] = 8.87, *p* < 0.01, for the test against zero).

There was also a significant main effect of task repetition, *F*(1, 35) = 201.02, *p* < .01, partial *η*^2^ = 0.86, and a significant task repetition × response repetition interaction, *F*(1, 35) = 39.96, *p* < 0.01, partial *η*^2^ = 0.53. Responses were faster if the task repeated (RT = 593 ms) than when it switched (RT = 651 ms). Also, under task-repetition conditions, there was an advantage of 27 ms (*t*[35] = 6.32, *p* < 0.01, for the test against zero) for response repetitions compared to response switches. The response repetition benefit turned into a − 19-ms cost (*t*[35] = − 3.72, *p* < 0.01, for the test against zero) under task-switching conditions; *t*(35) = 6.32, *p* < 0.01, for the difference between the response-repetition effects.

#### Complementary analyses of reaction times

An ANOVA of log-transformed RTs yielded qualitatively the same results.

To not only rely on the null effects concerning the missing higher-order interactions involving *n*-1 validity, *n*-validity, task repetition, and/or response repetition, we also conducted post-hoc analysis split-up based on task and response repetition/switch. *T* tests confirmed that the *n*-validity effect following *n*-1 valid trials was stronger than the *n*-validity effect following *n*-1 invalid trials in both task-repetition conditions, *t*(35) = 3.46, *p* < 0.01, and task-switching conditions, *t*(35) = 1.70, *p* < 0.05, one-sided. *T* tests also showed that *n*-validity effects were smaller following *n*-1 invalid than *n*-1 valid trials for trial-to-trial response repetitions, *t*(35) = 2.73, *p* < 0.05, and response switches, *t*(35) = 2.11, *p* < 0.05.

It is also known that conflict sequence effects can be due to spatial SR relations: one finds more facilitation by congruent responses (to the side of the target) compared to incongruent responses (to the opposite side of the target) following *n*-1 SR congruent than *n*-1 SR incongruent trials (Stürmer, Leuthold, Soetens, Schröter, & Sommer, 2002). On request of one of the reviewers, we tested if such SR congruence sequence effects complicated the picture, we conducted a further ANOVA with *n*-1 SR congruence and *n*-SR congruence as additional independent variables, and with data collapsed across steps of the variables task and task repetition. (Collapsing was necessary as the additional independent variables were not originally planned for, such that too few data per cells would have remained if all of the above independent variables plus the two additional independent variables would have been included.) This ANOVA yielded the same *n*-validity effect, *F*(1, 35) = 80.62, *p* < 0.01, partial *η*^2^ = 0.70, and *n*-1 validity × *n*-validity interaction *F*(1, 35) = 14.36, *p* < 0.01, partial *η*^2^ = 0.29, as the ANOVA above, plus significant main *n*-SR congruence, *F*(1, 35) = 26.71, *p* < 0.01, partial *η*^2^ = 0.43, and *n*-1 SR congruence effects, *F*(1, 35) = 9.99, *p* < 0.01, partial *η*^2^ = 0.22, as well as an interaction between these two variables, *F*(1, 35) = 64.63, *p* < 0.01, partial *η*^2^ = 0.65. The latter demonstrated the known SR congruence sequence effect, with larger SR congruence effects (SR incongruent minus SR congruent performance) following *n*-1 SR congruent trials (38 ms), *t*(35) = 7.95, *p* < 0.01, than *n*-1 SR incongruent trials (7 ms), *t*(35) = 2.00, *p* < 0.05, one-sided; *t*(35) = 9.22, *p* < 0.01, for the difference between the two congruence effects. Importantly, there were no significant higher-order interactions involving (1) the *n*-1 validity × *n*-validity interaction and either (2) *n*-1 SR congruence, (3) *n*-SR congruence, or (4) the *n*-1 SR congruence × *n*-SR congruence interaction, *F*s < 2.50, all *p*s > 0.12.

#### Error rates

The same ANOVA as was originally performed on correct RTs was conducted on the arc-sine transformed error rates (ERs). It led to significant main effects of task repetition, *F*(1, 35) = 65.91, *p* < 0.01, partial *η*^2^ = 0.65, and response repetition, *F*(1, 35) = 15.68, *p* < 0.01, partial *η*^2^ = 0.31, as well as to a significant interaction between these two variables, *F*(1, 35) = 16.00, *p* < .01, partial *η*^2^ = 0.31. ERs were lower in task-repetition (ER = 2.1%) than task-switching conditions (ER = 5.4%), and they were lower in response-repetition (ER = 2.9%) than response-switching conditions (ER = 4.6%). The interaction reflected an advantage of 3.5% (*t*[35] = 4.19, *p* < 0.01, for the test against zero) less errors under response-repetition conditions compared to response switches under task-switching conditions that reverted into a nonsignificant numerical advantage of 0.2% (*t* < 1.00) less errors for response switches compared to response repetitions under task-repetition conditions, *t*(35) = 4.00, *p* < 0.01, for the difference between the response-repetition effects. This is the opposite pattern of that found in the RT analysis and indicates that the latter could partly be due to a speed-accuracy trade-off. In addition, there was also a significant *n*-1 validity × response repetition interaction, *F*(1, 35) = 6.17, *p* < 0.05, partial *η*^2^ = 0.15, that reflected a stronger response-repetition advantage over response-switching conditions of 2.1% (*t*[35] = 4.40, *p* < 0.01, for the test against zero) following an *n*-1 valid trial than of 1.3% (*t*[35] = 3.00, *p* < 0.01, for the test against zero) following an *n*-1 invalid trial, *t*(35) = 2.48, *p* < 0.05, for the difference between the response-repetition effects. Finally, there was a significant four-way interaction between the variables task, response repetition, task repetition, and *n*-1 validity, *F*(1, 35) = 4.15, *p* < 0.05, *η*^2^ = 0.10. Importantly, there was no significant *n*-1 validity × *n*-validity interaction in the error rates, and there was also no higher-order interaction involving this two-way interaction (see also Table 1).

**Table 1:** Error rates in percent as a function of trial-to-trial task repetition (repetition vs. switch), trial-to-trial response repetition (repetition vs. switch), validity in the preceding trial (*n*-1 valid, vs. *n*-1 invalid) and validity in trial *n*, together with the corresponding *n-*validity effects (invalid error rate minus valid error)

Task	Response	*n*-1 val.			*n*-1 inv.		
		Valid	Invalid	Effect	Valid	Invalid	Effect
rep.	rep.	1.8	2.4	0.6	2.0	2.7	0.7
	swi.	1.9	2.3	0.4	2.1	1.8	−0.3
swi.	rep.	3.2	3.3	0.1	3.6	4.3	0.7
	swi.	6.9	7.9	1.0	6.6	7.2	0.6

Data from Experiment 1

*rep*. repetition, *swi*. switch, *val*. valid, *inv*. invalid

### Discussion

We tested if the validity sequence effect was restricted to task and/or response repetitions and found that this was not the case. In the overall ANOVA, the validity sequence effect did not significantly interact with either task or response repetition. In addition, post-hoc tests confirmed significant validity sequence effects under task-repetition and task-switching conditions as well as under response-repetition and response-switching conditions. According to the priming explanation, the validity sequence effect should have been restricted to task repetitions in which full repetitions could have made a difference between the *n*-validity effects following *n*-1 valid vs. *n*-1 invalid trials.

Relatedly, according to the event-file coding explanation, the validity sequence effect should have only obtained under task and response repetitions where full event-file repetitions could have affected the *n*-validity effect following *n*-1 valid compared to *n*-1 invalid trials. However, the two-way interaction of *n*-1 validity and *n*-validity was not involved in any significant higher-order interactions with task repetition and/or response repetition in the omnibus ANOVA, and the validity sequence effect was also found when we restricted our analysis to response-switching trials. These findings do not lend much support either to the priming or event-coding explanations, and thus leave room for the claim that some of the validity sequence effect was independent of inter-trial feature repetitions.

This repetition-independent validity sequence effect probably reflected processes taking place prior to target processing. This can be concluded from the facts that there were task repetition and response repetition effects, both of which depended on the target identity. As these two effects did not fully account for the validity sequence effect, it is likely that to some extent only the target-preceding cue processing was modulated by trial *n*-1’s cue validity. The fact that the validity sequence effect was due to target-preceding cue processing is better in line with an explanation in the form of more processing of expected helpful cues following an *n*-1 valid than invalid trial (cf. Gratton et al., 1992) or with more shielding against capture of attention by the cues following an *n*-1 invalid than valid trial (cf. Botvinick et al., 2001) than with a priming or event-file coding explanation.

The fact that the validity sequence effect was found with response switches, too, is particularly interesting in light of the observation that the validity effect itself was significantly stronger under response-repetition than response-switching conditions. Together, these observations indicate that part of the validity effect was not due to cue-driven pre-target processing alone, such as latency priming of the targets (Scharlau, 2002), and instead was probably due to post-target processing, such as influences of the cues on decisions concerning from which location to report (Shiu & Pashler, 1994).

The fact that there were no significant interactions between the validity sequence effect and trial-to-trial task and response repetitions in the omnibus ANOVA seems to be at variance with some of the literature suggesting that capture of attention by a stimulus can be primed by attending to a similar stimulus on the preceding trial (Awh, Belopolsky, & Theeuwes, 2012; Belopolsky, Schreij, & Theeuwes, 2010; Maljkovic & Nakayama, 1994). It also seems to be at variance with studies showing that inter-trial priming of attention does depend on (and, thus, interacts with) trial-to-trial response repetitions (Hillstrom, 2000; Huang, Holcombe, & Pashler, 2004). On closer inspection, there were a few differences between prior studies and the current Experiment 1 that might explain the absence of the critical interactions in the present experiment. First, some earlier studies looked at validity effects as a function of feature priming of cues in trial *n* by (1) features of the targets in trial *n*-1 or by (2) target-preceding primes in the same trial (Ansorge & Becker, 2012; Belopolsky et al., 2010; Folk & Remington, 2008). This is different from the present situation in which the cues were of a different color than all of the targets and, thus, inter-trial priming of capture by target-to-cue priming was ruled out. Second, studies that showed an influence of trial-to-trial response repetitions used visual search tasks in which attentional capture by the targets rather than by the cues was investigated (Hillstrom, 2000; Huang et al., 2004). These important differences were the reason for our second experiment, in which we increased the similarity to prior studies by allowing inter-trial priming of cue features by *n*-1 target features in at least some of the trials.

## Experiment 2

### Methods

#### Participants

Twenty-four participants (10 female, 14 male, aged 19 to 39 years; *M*_Age_ = 24.4 years) participated. Informed consent was obtained. All participants had normal or corrected-to-normal vision and intact color vision.

#### Stimuli and procedure

**Fig. 3:**
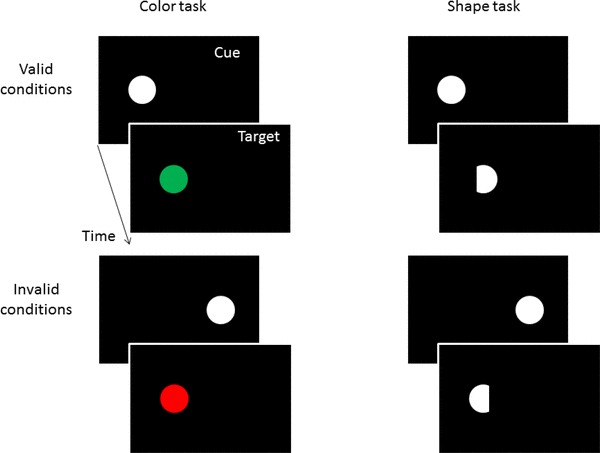
Schematic examples of the sequence of events in valid and invalid trials of the color task (on the left) and of the shape task (on the right) in Experiment 2. The only difference concerns the colors of the targets in the shape task that now fostered the task association of the cues with the shape task and that allowed inter-trial priming of cue colors by preceding target-shape colors. Stimuli are not drawn to scale

These were the same as before with two differences. First, the targets in the shape task were now presented in white, just as the cues (see Fig. 3). This would have allowed trial-to-trial priming of *n*-validity effects by color-similar *n*-1 shape targets but not following the red and green *n*-1 color targets.

### Results

#### Reaction times

**Fig. 4:**
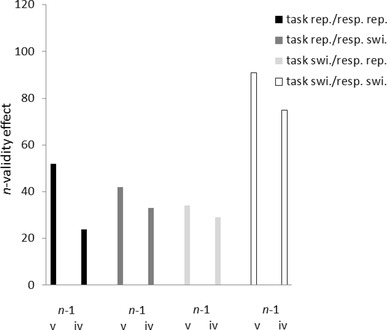
*n*-validity effects (*n*-invalid reaction time [RT] minus *n*-valid RT; in ms) on the abscissa as a function of *n*-1 validity (*n*-1 valid vs. *n*-1 invalid) on the ordinate, and of trial-to-trial task repetition (repetition: black and dark gray bars vs. switch: light gray and white bars) and response repetition (repetition: black and light gray bars vs. switch: dark gray and white bars). Data from Experiment 2. *v* valid, *i* invalid, *rep*. repetition, *swi*. switch

Trials following an error in *n*-1 (2.3%) or with RTs below 100 ms or above 1 s (another 1.1%) were excluded. An ANOVA of the correct RTs analogous to Experiment 1 was conducted. It led to a significant main effect of *n*-validity, *F*(1, 23) = 134.53, *p* < 0.01, partial *η*^2^ = 0.85, and to a significant *n*-validity × *n*-1 validity interaction, *F*(1, 23) = 89.51, *p* < 0.01, partial *η*^2^ = 0.80. RTs were propelled under valid (RT = 497 ms) compared to invalid (RT = 579 ms) conditions, and this validity effect (invalid RT minus valid RT) was stronger following *n*-1 valid (validity effect = 94 ms, *t*[23] = 12.82, *p* < 0.01, for the test against zero) than *n*-1 invalid (validity effect = 71 ms, *t*[23] = 10.01, *p* < 0.01, for the test against zero) trials, *t*(23) = 9.46, *p* < 0.01, for the difference between the validity effects (see also Fig. 4).

This time, however, the *n*-validity × *n*-1 validity interaction was involved in a higher-order interaction with response repetition, *F*(1, 23) = 7.17, *p* < 0.05, partial *η*^2^ = 0.24, and a five-way interaction between all variables, *F*(1, 23) = 5.30, *p* < 0.05, partial *η*^2^ = 0.19. To understand these interactions, we compared the validity effects following *n*-1 valid trials with the validity effects following *n*-1 invalid trials, separately for each step of the variables task, task repetition, and response repetition. For the white shape targets, the validity sequence effect was most pronounced if the task and the response repeated, and it was the weakest if the task repeated but the response switched (see Table 2). This was also reflected in a follow-up analysis of only the shape task, in which the *n*-1 validity × *n*-validity × response repetition interaction was significant, *F*(1, 23) = 6.24, *p* < 0.05, partial *η*^2^ = 0.21. In the follow-up analysis of only the color task, in contrast, we found an *n*-1 validity × *n*-validity interaction, *F*(1, 23) = 35.46, *p* < 0.01, partial *η*^2^ = 0.61, but the three-way interaction including response repetition was not significant, *F*(1, 23) = 2.98, *p* < 0.10, *η*^2^ = 0.12. Here, numerically the validity sequence effect was the strongest if the task switched and the response repeated, and it was the weakest if the task and the response repeated (see Table 2).

**Table 2:** *n*-validity effects as a function of task (c = color; s = shape), task repetition (task rep; r = trial-to-trial repetition; s = trial-to-trial switch), response repetition (resp rep; r = trial-to-trial repetition; s = trial-to-trial switch), and trial *n*-1 validity (*n*-1 v = *n*-1 valid; *n*-1 i = *n*-1 invalid), as well as the *t* values of *t* tests (all with 23 *df*) of the *n*-validity effects against zero (*t* [vs. 0]) and of the *n*-validity effects following *n*-1 valid vs. *n*-1 invalid trials (*t*)

Task	Task rep	Resp rep	*n*-1 v (ms)	*t* (vs. 0)	*n*-1 i	*t* (vs. 0)	*t*
c	r	r	88	10.49**	71 ms	8.01**	2.46*
c	r	s	93	11.20**	76 ms	8.84**	3.23**
c	s	r	106	13.18**	68 ms	7.23**	6.03**
c	s	s	91	12.80**	76 ms	10.56**	3.40**
s	r	r	99	11.26**	60 ms	6.53**	4.89**
s	r	s	88	9.40**	74 ms	8.98**	1.89
s	s	r	93	11.40**	67 ms	7.83**	4.60**
s	s	s	90	10.96**	74 ms	9.99**	4.00**

Data from Experiment 2

*Significant at *p* < 0.05

**Significant at *p* < 0.01

To note, however, with one exception, significant validity sequence effects were found in all of the comparisons (see Table 2, last column). The exception was task repetitions but response switches in the shape task, in which case, there was only a numerically stronger *n*-validity effect following *n*-1 valid than *n*-1 invalid trials.

Besides these findings, which were of primary interest, we also observed two significant three-way interactions, a task × task repetition × response repetition interaction, *F*(1, 23) = 4.41, *p* < 0.05, partial *η*^2^ = 0.16, and an *n*-1 validity × task repetition × response repetition interaction, *F*(1, 23) = 4.37, *p* < 0.05, partial *η*^2^ = 0.16. If the shape task was repeated, there was a small cost incurred by a response switch of − 8 ms, *t*(23) = − 2.76, *p* < 0.05, that was lacking (2 ms) if the participants switched from an *n*-1 color task trial to an *n*-shape task trial, *t*(23) = 1.79, *p* = 0.09, for the difference between the response-switching costs. If anything, this pattern numerically reversed with the *n*-color task trials, in which task repetitions created numerically smaller (but nonsignificant) response-switching costs (of − 1 ms) than task switches (− 5 ms), *t*(23) = − 1.24, *p* = 0.23. An analogous pattern was created by *n*-1 validity. Following *n*-1 valid trials, in task-repetition trials, there was a response switching cost of − 8 ms that was absent (1 ms) in task-switching trials, *t*(23) = 1.91, *p* = 0.07, for the difference between the response-switching costs. Numerically, this pattern was reversed following *n*-1 invalid trials, with response switching costs of − 2 ms in task-repetition trials and of − 5 ms in task-switching trials, *t*(23) = − 1.23, *p* = 0.23, for the difference between the response-switching costs.

Of further note, clear-cut evidence of an influence of inter-trial priming of attention capture by cues with a color similar to *n*-1 target colors was missing. This priming of attention would have obtained following *n*-1 shape-task trials only: it should have led to a significant task × task repetition × *n*-validity interaction. Besides the above five-way interaction that included this three-way interaction, there was no clear evidence of inter-trial priming of attention capture: neither the three-way interaction itself nor any of the four-way interactions including it was significant, all nonsignificant *F*s < 2.60, all *p*s > 0.11.

#### Complementary analyses of reaction times

An ANOVA of log-transformed RTs yielded qualitatively very similar results, with the exception of a theoretically meaningful significant *n*-1 validity × *n*-validity × response repetition interaction, *F*(1, 23) = 7.18, *p* < 0.05, partial *η*^2^ = 0.24, that was missing in the initial ANOVA. The congruence sequence effect was, however, significant in ANOVAs split-up for response-repetition conditions, *F*(1, 23) = 63.29, *p* < 0.01, partial *η*^2^ = 0.73, and, though somewhat weaker, for response-switching conditions, *F*(1, 23) = 23.94, *p* < 0.01, partial *η*^2^ = 0.51.

Similar to Experiment 1, an ANOVA with *n*-1 SR congruence and *n*-SR congruence instead of task and task repetition as independent variables yielded a significant main effect of *n*-validity, *F*(1, 23) = 134.30, *p* < 0.01, partial *η*^2^ = 0.85, and a significant *n*-1 validity × *n*-validity interaction *F*(1, 23) = 109.69, *p* < 0.01, partial *η*^2^ = 0.83, plus significant main *n*-SR congruence, *F*(1, 23) = 4.39, *p* < 0.05, partial *η*^2^ = 0.16, and *n*-1 SR congruence, *F*(1, 23) = 22.05, *p* < 0.01, partial *η*^2^ = 0.49, effects, as well as an interaction between these two variables, *F*(1, 23) = 99.29, *p* < 0.01, partial *η*^2^ = 0.81, with larger SR congruence effects following *n*-1 SR congruent trials (31 ms), *t*(23) = 5.68, *p* < 0.01, than *n*-1 SR incongruent trials (− 10 ms), *t*(23) = − 1.93, *p* = 0.07; *t*(23) = 9.96, *p* < 0.01, for the difference between the two congruence effects.

Different from Experiment 1, there was a significant higher-order interaction involving (1) the *n*-1 validity × *n*-validity interaction and (2) the *n*-1 SR congruence × *n*-SR congruence interaction, *F*(1, 23) = 8.91, *p* < 0.01, partial *η*^2^ = 0.28. To test, if the validity sequence effect depended on some particular confounding side condition in terms of the SR congruence sequence effect, we ran ANOVAs split-up for the four combinations of *n*-1 SR congruence and *n*-SR congruence, and confirmed significant *n*-1 validity × *n*-validity interactions in all of them, all significant *F*s > 12.00, all *p*s < 0.01, see also Table 3.

**Table 3:** *n*-validity reaction time (RT) effects (*n*-invalid RT minus *n*-valid RT) of Experiment 2 as a function of *n*-1 validity (*n*-1 valid vs. *n*-1 invalid), *n*-1 SR congruence (*n*-1 congruent vs. *n*-1 incongruent) and *n*-SR congruence (*n*-congruent vs. *n*-incongruent), as well as the corresponding *F* values of the *n*-1 validity × *n*-validity interactions

		*n*-1 val.	*n*-1 inv.	*F*(1, 23)
*n*-1 SR cong.	*n*-SR cong.	86	62	51.64**
*n*-1 SR cong.	*n*-SR inc.	103	81	34.79**
*n*-1 SR inc.	*n*-SR cong.	89	75	12.50**
*n*-1 SR inc.	*n*-SR inc.	99	68	79.47**

Data from Experiment 2

*n*-1 preceding trial, *n* current trial, *SR* stimulus–response, *cong*. congruent, *inc*. incongruent, *val*. valid, *inv*. invalid

**Significant at *p* < 0.01

The ANOVA also replicated the significant *n*-1 validity × *n*-validity × response repetition interaction, *F*(1, 23) = 6.96, *p* < 0.05, partial *η*^2^ = 0.23, that we found in the initial analysis. As for the initial analysis, a significant validity sequence effect was present in response-repetition, *F*(1, 23) = 62.55, *p* < 0.01, partial *η*^2^ = 0.73, and response-switch conditions, *F*(1, 23) = 32.28, *p* < 0.01, partial *η*^2^ = 0.58, but it was reduced in the latter. There was also a significant *n*-1 SR congruence × *n*-SR congruence × response repetition interaction, *F*(1, 23) = 5.44, *p* < 0.05, partial *η*^2^ = 0.19. Analogously to Experiment 1 and to the influence of response repetitions on the validity sequence effect in the present experiment, there was a stronger *n*-1 SR congruence × *n*-SR congruence interaction among the response repetitions, *F*(1, 23) = 78.42, *p* < 0.01, partial *η*^2^ = 0.77, than among the response switches, *F*(1, 23) = 69.48, *p* < 0.01, partial *η*^2^ = 0.75.

In addition, there was a significant *n*-1 SR congruence × *n*-SR congruence × *n*-validity interaction, *F*(1, 23) = 5.83, *p* < 0.05, partial *η*^2^ = 0.20, that was further qualified by the four-way interaction that we have discussed above (see Table 3). By collapsing across *n*-1 valid and *n*-1 invalid conditions in Table 3, it can be seen that the lowest *n*-validity effect resulted in *n*-1 SR congruent/*n*-SR congruent conditions and that the highest *n*-validity effect was found in *n*-1 SR congruent/*n*-SR incongruent conditions. A reason could be that *n*-1 SR congruent/*n*-SR congruent conditions facilitated responding the most, thereby potentially undermining any facilitation by *n*-valid cues (and hence an *n*-validity effect) through a bottom effect in the overall RTs alone. In contrast, enticing the participants to the processing of irrelevant stimulus positions in *n* by an *n*-1 SR congruent trial was probably most harmful for the responses when an *n*-SR incongruent trial followed. In this situation, participants might have relied the most on visuo-spatial attention.

Finally, *n*-1 SR congruence significantly interacted with response repetition, *F*(1, 23) = 26.36, *p* < 0.01, partial *η*^2^ = 0.53: for unknown reasons, following an *n*-1 SR congruent trial, there was no advantage for response repetitions compared to response switches (− 3 ms), *F*(1, 23) = 1.00, *p* = 0.33, partial *η*^2^ = 0.04. This was different for response repetitions compared to response switches from an *n*-1 SR incongruent trial (10 ms), *F*(1, 23) = 7.74, *p* < 0.05, partial *η*^2^ = 0.25. As we will see below, this effect could have (partly) reflected a speed-accuracy trade-off.

#### Error rates

An ANOVA of the arc-sine transformed error rates, with the variables *n*-1 validity, n-validity, task, task repetition, and response repetition, yielded significant main effects of validity, *F*(1, 23) = 31.22, *p* < 0.01, partial *η*^2^ = 0.58, response repetition, *F*(1, 23) = 11.70, *p* < 0.01, partial *η*^2^ = 0.34, and a significant interaction between these variables, *F*(1, 23) = 6.65, *p* < 0.05, partial *η*^2^ = 0.22. Error rates were lower in valid (1.3%) than invalid (2.4%) conditions, and under trial-to-trial response repetition (1.4%) than switch (2.3%) conditions. In addition, the validity effect was more pronounced under response-switching (1.43%, *t*[23] = 5.10, *p* < 0.01) than under response-repetition (0.5%, *t*[23] = 1.88, *p* = 0.07) conditions, *t*(23) = 2.58, *p* < 0.01, for the difference between the validity effects. As in the RTs, there was also a three-way task × task repetition × response repetition interaction, *F*(1, 23) = 5.76, *p* < 0.05, partial *η*^2^ = 0.20. In contrast to the RT effect, it reflected a weaker response-switching cost where the shape task repeated (0.1%) than if the participants switched from an *n*-1 color task trial to an *n-*shape task trial (1.3%), *t*(23) = − 2.10, *p* < 0.05. This pattern was reversed for color task repetitions, in which the response-switching costs under task repetition conditions (1.3%) numerically but not significantly exceeded the response-switching costs under task-switching (0.8%) conditions, *t*(23) = 1.21, *p* = 0.24, for the difference between the response-switching costs.

Again, inter-trial priming of capture was not unambiguously supported, as neither the task × task repetition × *n*-validity interaction nor any higher-order interaction including it became significant, all nonsignificant *F*s < 1.00, see also Table 4.

**Table 4:** Error rates in percent as a function of trial-to-trial task repetition (repetition vs. switch), trial-to-trial response repetition (repetition vs. switch), validity in the preceding trial (*n*-1 valid, vs. *n*-1 invalid) and validity in trial *n*, together with the corresponding *n-*validity effects (invalid error rate minus valid error rate)

Task	Response	*n*-1 val.			*n*-1 inv.		
		Valid	Invalid	Effect	Valid	Invalid	Effect
rep.	rep.	1.2	1.8	0.6	1.2	1.6	0.4
	swi.	1.4	2.9	1.5	1.5	2.8	1.3
swi.	rep.	1.1	1.4	0.3	1.2	1.9	0.7
	swi.	1.4	3.5	2.1	1.7	3.2	1.5

Data from Experiment 2

*rep*. repetition, *swi*. switch, *val*. valid, *inv*. invalid

### Discussion

We found validity sequence effects even where the task and the response switched from trial to trial, in both the color task and the shape task. The only condition in which the validity sequence effect broke down was in the shape task if the task repeated but the response was switched from the previous trial. This selective reduction in (or removal of) the validity sequence effect was neither predicted by the priming nor by the event-file coding explanation.

Yet, it is interesting to consider which factors might have been responsible for the newly emerging influences of trial-to-trial response repetitions on validity sequence effects that were missing in Experiment 1. The now stronger influence of inter-trial priming effects could have been due to the integration of the cues into the task and trial representations (Schumacher & Hazeltine, 2016). Whereas in Experiment 1, the cues were of a different color than all of the targets, in Experiment 2, the cues were of the same color as the shape targets. If the participants at least occasionally used target colors to decide about the current task, the white cue, which shared the color of the shape targets, would have served as a task cue (though non-predictively and, at times, misleadingly), unlike in Experiment 1. This could have opened the gate for its joint representation together with the targets and the responses, so that response-repetition influences now could have influenced the validity sequence effect to also bring about significant interactions in the omnibus ANOVA. This possibility was tested more systematically in Experiment 3.

However, independently of task set representations, it is also possible that a combination of between- and within-trial color similarities prompted the decrease of the validity sequence effect in response-switching conditions of shape-task repetition trials. First, inter-trial priming of cue color by *n*-1 target color was only possible following a shape-task trial, but, second, cue-to-target color similarity could have only fostered smooth integration of cue and target into one joint event file in a shape-task trial (Carmel & Lamy, 2014, 2015). This means that favorable conditions for cue-color processing *and* cue-target integration were jointly met only when the shape task repeated from *n*-1 to *n*. If this had reinforced joint representations of cue and target with further features, such as shape or response identity, into one joint event file, response switches could have also incurred rebinding costs under these special conditions. While this would allow explaining some variation of the size of the validity sequence effect by inter-trial priming or event coding regardless of task set representations, it is clear that, on the whole, this explanation could not account for all of the sequence effects that we found so far.

## Experiment 3

After Experiment 2, we discussed the possibility that the participants’ usage of peripheral cues as task cues could have gated task and response repetition influences on validity sequence effects (cf. Hazeltine et al., 2011). However, in Experiment 2 the results were not showing evidence of a straightforward priming account of the validity sequence effect either. Instead, the validity sequence effect was partly independent of feature repetitions across trials, especially in the color task.

To get a clearer picture of the putative role of cue-to-task associations as facilitators for priming contributions to validity sequence effects, we conducted Experiment 3, in which we used peripheral cues that were unique to each of two upcoming SR mappings and thus 100% predictive of these mappings. This scenario should encourage incorporation of the cues into different task sets, as the cues would reduce the uncertainty about the upcoming SR mapping and, thus, decrease task difficulty. This means that each of the two peripheral cues that we used was associated with only one particular task. In this way, each cue signaled a particular task and we thus strengthened the cue-to-task associations. If task-file theory is correct and cue-to-task associations gate influences of trial-to-trial priming on validity sequence effects, we should find stronger evidence of priming as the responsible factor for validity sequence effects in Experiment 3 than in Experiments 1 and 2. For example, a stronger link between cue and task could also foster associations between cues and task-specific targets so that inter-trial priming based on episodes consisting of cue and target, or based on events of cue, target and response becomes more likely.

### Methods

#### Participants

Twenty-three participants (21 female, 2 male, aged 18 to 41 years; *M*_Age_ = 23.1 years) participated. (Data from one additional participant were lost.) Informed consent was obtained. All participants had normal or corrected-to-normal vision and intact color vision.

#### Stimuli and procedure

Stimuli and procedure were the same as before, with the following exceptions. RTs and response identities were registered via keys #A, #S, #C, and #F (for half the participants) or #F, #G, #X, and #S (for the other half) of a QWERTZ computer keyboard in front of the participants. Keys #A and #S (and #S and #X) were operated with the left middle and index fingers, respectively; keys #C and #F (and #F and #G) with the right index and middle fingers, respectively.

**Fig. 5:**
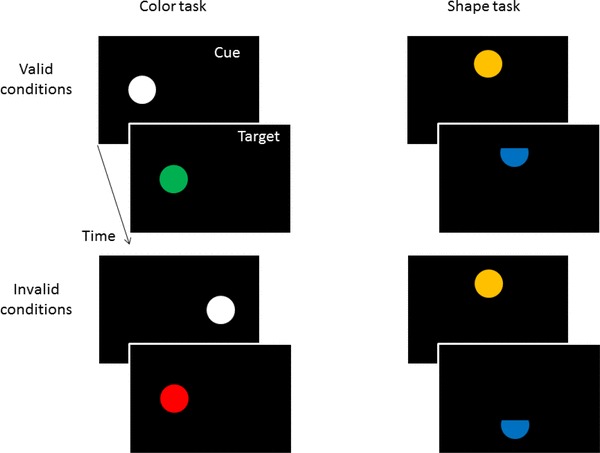
Schematic examples of the sequence of events in valid and invalid trials of the color task (on the left) and of the shape task (on the right) in Experiment 3. In contrast to Experiments 1 and 2, the tasks and cues were now more different. The two tasks differed in terms of the stimulus positions, cue colors, and responses (not depicted). Also, cues now signaled the different tasks. Stimuli are not drawn to scale

Again two tasks were used: a shape and a color task. To emphasize the differences between tasks and task-associated cues, three measures were taken. First, different hands were used for different tasks. Half the participants discriminated colors with the right hand, and shapes with the left hand, the other half got the reverse mapping. Second, different target and cue positions were used. Whereas cues and targets of the color task were shown on the horizontal axis (as before), the cues and targets of the shape task were presented on the vertical axis (see Fig. 5). (Eccentricity of all stimuli from screen center was the same as before.) Third, only in the color task, a white cue was used as before. In the shape task, however, the cue was brown (CIE color coordinates = 0.45/0.46) and of the same luminance as the other colors (see Fig. 5). With these three measures, we aimed to create grouping within task elements and minimize grouping between task elements (Adam, Hommel, & Umiltà, 2003), and thus enforce the cue-to-task and cue-to-target associations to the effect of more task and response priming influences on validity sequence effects.

Again, on each trial, unforeseeably for the participants one of two tasks was administered: the color or the shape task. In the color task, half the participants pressed right for red (#G or #S, depending on the hand used for color discrimination) and left for green targets (#F or #A). In the shape task, participants pressed the upper button for targets missing the top (#F or #S, depending on the hand used) and the lower button for targets missing the bottom (#C or #X).

### Results

#### Reaction times

One participant was excluded because her error rate exceeded 20% of all trials in task-switching conditions. Trials following an error in *n*-1 (4.6%) or with RTs below 100 ms or above 1 s (another 3.3%) were excluded. Where the sphericity assumption was violated, degrees of freedom were adjusted by Greenhouse-Geisser ε and corrected *p* values reported.

We conducted a repeated-measurements analysis of variance (ANOVA) of mean correct RTs, with variables (1) *n*-validity (*n*-valid; *n*-invalid), (2) *n*-1 validity (*n*-1 valid; *n*-1 invalid), (3) task (color task; shape task), and (4) trial-to-trial task/response repetition (task/response repetition; task repetition/response switch; task switch). (In contrast to Experiments 1 and 2, there were no conditions in which the task switched and the response repeated, so that there were no two orthogonal variables of task repetition and response repetition but rather only one fused three-step variable of task/response repetition.)

**Fig. 6:**
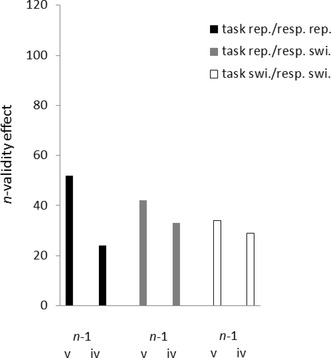
*n*-validity effects [*n*-invalid reaction time (RT) minus *n*-valid RT; in ms] on the abscissa as a function of *n*-1 validity (*n*-1 valid vs. *n*-1 invalid) on the ordinate, and of trial-to-trial task/response repetition (task and response repetition: black bars vs. task repetition/response switch: light gray bars vs. task and response switch: white bars). Data from Experiment 3. *v* valid, *i* invalid, *rep*. repetition, *swi*. switch

Besides a significant main effect of *n*-validity, *F*(1, 22) = 110.29, *p* < 0.01, partial *η*^2^ = 0.82, and a significant *n*-validity × *n*-1 validity interaction, *F*(1, 22) = 33.13, *p* < 0.01, partial *η*^2^ = 0.60, there was a significant three-way *n*-validity × *n*-1 validity × task/response repetition interaction, *F*(1, 22) = 3.45, *p* < 0.05, partial *η*^2^ = 0.14. See also Fig. 6. In general, participants were faster under *n*-valid (556 ms) than under *n*-invalid (591 ms) conditions. This *n*-validity effect was stronger following *n*-1 valid (*n*-validity effect = 43 ms; *t*[22] = 11.15, *p* < 0.01, for the test against zero) than following *n*-1 invalid (*n*-validity effect = 29 ms; *t*[22] = 7.75, *p* < 0.01, for the test against zero) trials, *t*(22) = 5.76, *p* < 0.01, for the difference between the *n*-validity effects. However, splitting up the data for the steps of the variable task/response repetition, it became clear that there was only a significant validity sequence effect when task *and* response repeated, where validity effects were again stronger following *n*-1 valid (*n*-validity effect = 52 ms) than *n*-1 invalid (*n*-validity effect = 24 ms) trials, *t*(22) = 3.94, *p* < 0.01, for the difference between the *n*-validity effects. In contrast, there were only nonsignificant numerical tendencies towards a validity sequence effect under task repetition/response switching conditions, where the *n*-validity effect was 42 ms following *n*-1 valid trials, and 33 ms following *n*-1 invalid trials, *t*(22) = 1.70, *p* = 0.10, for the difference between the *n*-validity effects, and under task-switching conditions, where the *n*-validity effect was 34 ms following *n*-1 valid trials, and 29 ms following *n*-1 invalid trials, *t* < 1.00, for the difference between the two *n*-validity effects.

Besides these effects of greatest interest, we found significant main effects of task and task/response repetition. Participants were faster in the color task (558 ms) than in the shape task (589 ms), and if the task and response repeated (538 ms) than if only the task repeated (566 ms) or if neither task nor response repeated (616 ms), all comparisons are significant at *p* < 0.01. The variable task also interacted significantly with *n*-validity, *F*(1, 22) = 12.18, *p* < 0.01, partial *η*^2^ = 0.36. This was due to a weaker *n*-validity effect in the color task (*n*-validity effect = 25 ms) than in the shape task (*n*-validity effect = 46 ms). There was also a two-way interaction of task × task/response repetition, *F*(2, 44) = 71.96, *p* < 0.01, partial *η*^2^ = 0.77, and a three-way interaction of task × *n*-1 validity × task/response repetition, *F*(2, 44) = 4.85, *p* < 0.05, partial *η*^2^ = 0.18. To understand the interactions, we compared response-switching costs in trial-to-trial repetitions (calculated as task-repetition/response-switching RT minus task-repetition/response-repetition RT) to task-switching costs for trial-to-trial response switches (calculated as task-switching/response-switching RT minus task-repetition/response-switching RT), either as a function of task (for the two-way interaction) or of task and *n*-1 validity (for the three-way interaction). Whereas in the color task the response-switching costs during trial-to-trial task repetitions (34 ms, *t*[22] = 6.51, *p* < 0.01, for a test against zero) were significantly smaller than the task-switching costs for trial-to-trial response switches (77 ms, *t*[22] = 10.28, *p* < .01, for a test against zero), *t*(22) = 4.77, *p* < 0.01, for the difference between the switching costs in the color task, in the shape task, the response switching costs during trial-to-trial task repetitions (22 ms, *t*[22] = 4.84, *p* < 0.01) and the task-switching costs during trial-to-trial response switches (22 ms, *t*[22] = 3.90, *p* < 0.01) were of about the same size, *t* < 1.00, for the difference between the switching costs in the shape task. Whatever the exact reason for the three-way interaction, as can be seen from Table 5, the pattern of higher task-switching than response-switching costs in the color task but more similar task-switching and response-switching costs in the shape task held, regardless of the level of *n*-1 validity.

**Table 5:** Response-switching costs (RSC) for trial-to-trial task repetitions (calculated as task-repetition/response-switching RT minus task-repetition/response-repetition RT) and task-switching costs (TSC) for trial-to-trial response switches (calculated as task-switching/response-switching RT minus task-repetition/response-switching RT) as a function of task (c = color; s = shape), and trial *n*-1 validity (*n*-1 v = *n*-1 valid; *n*-1 i = *n*-1 invalid), as well as the *t* values of *t* tests (all with 22 *df*) of the switching costs against zero (*t* [vs. 0]) and of the response-switching costs vs. the task-switching costs (*t*)

task	*n*-1 val.	RSC (ms)	*t* (vs. 0)	TSC (ms)	*t* (vs. 0)	*t*
c	*n*-1 v	31	4.54**	74	9.89**	4.31**
c	*n*-1 i	38	7.39**	80	10.04**	4.30**
s	*n*-1 v	24	4.37**	23	4.56**	ns
s	*n*-1 i	19	4.13**	20	2.86**	ns

Note that there were no task-switching/response-repetition conditions in Experiment 3 because responses in different tasks were given with different hands

*ns* not significant

*Significant at *p* < 0.05

#### Complementary analyses of reaction times

The ANOVA of the log-transformed RTs did not yield any differences compared to the initial ANOVA.

The ANOVA with the independent variables *n*-1 SR congruence and *n*-SR congruence, with data collapsed across tasks, replicated the *n*-validity effect, *F*(1, 22) = 95.28, *p* < 0.01, *η*^2^ = 0.81, the task/response repetition effect, *F*(2, 44) = 88.70, *p* < 0.01, *η*^2^ = 0.80, the *n*-1 validity × *n*-validity interaction, *F*(1, 22) = 26.95, *p* < 0.01, *η*^2^ = 0.55, and the *n*-1 validity × *n*-validity × task/response repetition interaction, *F*(2, 22) = 5.26, *p* < 0.01, η^2^ = 0.19. This ANOVA yielded evidence of the following additional significant effects: *n*-SR congruence, *F*(1, 22) = 12.26, *p* < 0.01, *η*^2^ = 0.36, and *n*-1 SR congruence, *F*(1, 22) = 11.37, *p* < 0.01, *η*^2^ = 0.34, and several interactions (see below). Responses were faster in *n*-SR congruent (566 ms) than *n*-SR incongruent conditions (581 ms), and following an *n*-1 SR congruent trial (571 ms) compared to an *n*-1 SR incongruent trial (575 ms). Again, there was an *n*-1 SR congruence × *n*-SR congruence interaction, *F*(1, 22) = 123.09, *p* < 0.01, *η*^2^ = 0.85. It reflected a stronger *n*-SR congruence effect following *n*-1 SR congruent trials (36 ms), *t*(22) = 6.99, *p* < 0.01, than following *n*-1 SR incongruent (− 6 ms), *t*(22) = − 1.47, *p* = 0.16; *t*(22) = 11.10, *p* < 0.01, trials, for the difference between the two *n*-SR congruence effects. Four further three-way interactions, of *n*-1 validity × task/response repetition × *n*-SR congruence, *F*(2, 44) = 3.55, *p* < 0.05, *η*^2^ = 0.14, of *n-*validity × task/response repetition × *n*-SR congruence, *F*(2, 44) = 7.02, *p* < 0.01, *η*^2^ = 0.24, of *n-*validity × *n*-1 SR congruence × *n*-SR congruence, *F*(1, 22) = 5.94, *p* < 0.05, *η*^2^ = 0.21, and of task/response repetition × *n*-1 SR congruence × *n*-SR congruence, *F*(2, 44) = 71.91, *p* < 0.01, *η*^2^ = 0.77, were all qualified by an almost significant five-way interaction, *F*(2, 44) = 3.14, *p* = 0.053, *η*^2^ = 0.13, and are only analyzed and discussed for their implications for the validity-sequence effect. As can be seen from Table 6, when task and response repeated, the validity sequence effect was significant in *n*-1 SR congruent/*n*-SR congruent and in *n*-1 SR incongruent/*n*-SR incongruent conditions, but it failed to become significant in *n*-1 SR congruent/*n*-SR incongruent and in *n*-1 SR incongruent/*n*-SR congruent conditions. This pattern would exactly match the predictions of a priming account where optimal priming should be observed the more features repeat from trial *n*-1 to trial *n*. In general, the observation that the validity sequence effect is more spurious when the response changes from *n*-1 to *n* would also be in line with the priming or event-file coding explanation, but the fact that here the validity sequence effect was strongest and only significant in *n*-SR incongruent trials following *n*-1 SR congruent trials would be unexpected under this perspective. Finally, the absence of a validity sequence effect for trial-to-trial task and response switches again perfectly aligns with an influence of inter-trial priming and/or event-filed coding contributions to the validity sequence effect.

**Table 6:** Given are the reaction time (RT) *n*-validity effects (*n*-invalid RT minus *n*-valid RT; in ms, with significance based on *t* tests against zero, with 22 *df*) as a function of *n*-1 validity (*n*-1 valid vs. *n*-1 invalid), trial-to-trial task/response repetition (both repeat vs. task repeats/response switches vs. both switch) *n*-1 stimulus–response (SR) congruence (*n*-1 SR congruent vs. *n*-1 SR incongruent), and *n*-SR congruence (*n*-congruent vs. *n*-incongruent), as well as the corresponding *F* values of *n*-1 validity × *n*-validity interactions of analyses of variance (with significance based on 1 and 22 degrees of freedom), split-up for steps of the independent variables task/response repetition, *n*-1 SR congruence, and *n*-SR congruence

Task/response	*n*-1 SR	*n*-SR	*n*-1 val.	*n*-1 inv.	*F*
rep./rep	cong.	cong.	40**	11	6.72*
	cong.	inc.	52**	31**	2.37
	inc.	cong.	53**	31*	3.08
	inc.	inc.	66**	10	28.26**
rep./swi	cong.	cong.	42**	44**	0.19
	cong.	inc.	53**	21*	10.79**
	inc.	cong.	54**	51**	0.14
	inc.	inc.	20*	17*	0.07
swi./swi	cong.	cong.	33**	35**	0.06
	cong.	inc.	28**	26**	0.06
	inc.	cong.	44**	34**	0.70
	inc.	inc.	33**	23**	1.73

Data from Experiment 3

*SR* stimulus-response, *val*. valid, *inv*. invalid, *rep*. trial-to-trial repetition, *swi*. trial-to-trial switch, *cong*. congruent, *inc*. incongruent

*Significant at *p* < 0.05

**Significant at *p* < 0.01

#### Error rates

An ANOVA of the arc-sine transformed error rates, with the variables task, task/response repetition, *n*-1 validity, and *n*-validity, confirmed the existence of a significant main effect of validity, *F*(1, 22) = 5.50, *p* < 0.05, partial *η*^2^ = 0.20. Error rate was lower in valid (2.8%) than invalid (3.5%) conditions. The *n*-validity × *n*-1 validity × task/response repetition interaction was almost significant, *F*(2, 44) = 3.06, *p* = 0.06, partial *η*^2^ = 0.12, but this time the only significant validity sequence effect was found in the task repetition/response switching conditions, in which the validity effect following an *n*-1 valid trial (1.6%) was significantly stronger than the validity effect following an *n*-1 invalid trial (− 0.4%), *t*(22) = 3.89, *p* < 0.01, see also Table 7. In contrast, in trial-to trial task/response repetition conditions and in trial-to-trial task-switching conditions, the validity effects following *n*-1 valid trials (1.1 and − 0.2%, respectively) were not significantly different from the validity effects following *n*-1 invalid trials (1.5 and 0.4%, respectively), both *t*s < 1.00 for the comparisons between the respective validity effects.

**Table 7:** Error rates in percent as a function of trial-to-trial task/response repetition (both repeat vs. task repeats and response switches vs. both switch), validity in the preceding trial (*n*-1 valid, vs. *n*-1 invalid) and validity in trial *n*, together with the corresponding *n-*validity effects (invalid error rate minus valid error rate)

Task	Response	*n*-1 val.			*n*-1 inv.		
		Valid	Invalid	Effect	Valid	Invalid	Effect
rep.	rep.	2.1	3.2	1.1	1.8	3.3	1.5
rep.	swi.	1.6	3.2	1.6	2.4	2.0	−0.4
swi.	rep.	4.4	4.2	−0.2	4.6	5.0	0.4

Data from Experiment 3

*rep*. repetition, *swi*. switch, *val*. valid, *inv*. invalid

Besides, there was a significant main effect of task/response repetition, *F*(2, 44) = 21.46, *p* < 0.01, partial *η*^2^ = 0.49. There were no costs associated with a response switch in trial-to-trial task repetitions: the error rates were 2.6% for task/response repetitions and 2.3% for task repetitions/response changes which were not significantly different from one another. However, task switches led to an error rate of 4.5%—that is, an additional cost compared to both of the task-repetition conditions (*p* < 0.01). There were also significant two-way task × *n*-validity, *F*(1, 22) = 5.84, *p* < 0.05, partial *η*^2^ = 0.21, and task × task/response repetition, *F*(2, 44) = 16.64, *p* < 0.01, partial *η*^2^ = 0.43, interactions. In the color task, there was no significant validity effect (0.2%, *t* < 1.00 for the difference from zero); only in the shape task, there was a significant validity effect (1.1%, *t*[22] = 2.90, *p* < 0.01 for the difference from zero); *t*(22) = 2.42, *p* < 0.05 for the difference between the validity effects. When we looked at the response switching costs under trial-to-trial task repetition conditions and at the task-switching costs under trial-to-trial response switching conditions, the only significant cost we found was a task-switching cost in the color task (3.9%, *t*[22] = 5.54, *p* < 0.01, for the difference from zero, all nonsignificant *t*s < 1.60, all *p*s > 0.14). This was also reflected in a significantly stronger task-switching (3.9%) than response-switching (0.3%) cost in the color task, *t*(22) = 4.75, *p* < 0.01, for the difference between these costs, but no significant difference between the task-switching (0.7%) and the response-switching (− 0.9%) in the shape task, *t*(22) = 1.66, *p* = 0.11, for the difference.

### Discussion

In Experiment 3, we fostered the differences between the tasks by presenting the stimuli of different tasks on different axes, using different hands, and using differently colored targets and cues for the two tasks. By the latter manipulation, we also fostered the association between different peripheral cues and tasks. Also, as each cue was shown on the same axis as the targets, the cue positions were 100% predictive of the upcoming tasks. With these manipulations, we found much stronger evidence for event-file coding contributions to validity sequence effects than in Experiments 1 and 2. With very few exceptions, such as a small validity sequence effect in the error rates under task-repetition/response-switching conditions, the validity sequence effect was restricted to task and response repetitions. These findings are in line with a role of task contexts for sequence effects (Schumacher & Hazeltine, 2016): analogous to previous priming studies that used different modalities for two intermixed tasks or only one modality per each of two different tasks (Hazeltine et al., 2011), in the present experiment using different cues to signal alternative tasks abolished across-task validity sequence effects. In addition, for these task-specific validity sequence effects, it was not necessary to use a cue of the same color as the subsequent (or preceding) target (as it had been the case in Experiment 2). In Experiment 3, cues were always of a color different from the following target, and so bottom-up feature similarities between cue and target are evidently not necessary to support task-specific validity sequence effects.

Given that in Experiment 3 we used three different features to promote discriminating between the tasks, it is unclear which features were responsible for the increased task specificity of the validity sequence effect. Two features of the cues discriminated between the tasks: their spatial positions on the vertical vs. on the horizontal axis, and their respective colors. The cues in Experiment 3 also differed from the cues in Experiments 1 and 2 by a further characteristic: only in Experiment 3, the cues were 100% informative of the upcoming tasks. Each of these three characteristics alone might have been sufficient to account for the stronger task specificity of the validity sequence effect in Experiment 3. In addition, the tasks also differed with respect to cue-unrelated characteristics: the axes on which the targets were presented, the colors and the shapes of the targets, and the responses required to discriminate between targets. Because target colors of Tasks 1 and 2 and target shapes of Tasks 1 and 2 were also different from one another in Experiments 1 and 2, but more evidence of cross-task and cross-response validity sequence effects was found in these experiments, these two characteristics were likely not responsible for the task and response specificity of the validity sequence effect. Also, on the basis of the present experiments, target positions alone could have been responsible for task specificity. However, this explanation is unlikely given the findings of Goller and Ansorge (2015). In their Experiment 2, these authors found validity sequence effects even when neither cue nor target position repeated from one trial to the next. In conclusion, however, it is still possible that at least different response sets or different position sets for the two alternative tasks alone could have prevented across-task validity sequence effects in the present experiment. However, what we can conclude based on Experiment 3 is that the validity sequence effect does not survive task-switching when the tasks are clearly distinguished.

We also found typical switching costs. Both trial-to-trial response switches and trial-to-trial task switches incurred an RT cost compared to trial-to-trial repetitions of tasks or responses. Interestingly, only in the color task, the task-switching costs exceeded the response-switching costs. The switching costs in the color task (77 ms) were also higher than that in the shape task (22 ms). Because in the current experiment the color task was easier than the shape task, this switching cost asymmetry is concordant with existing research in which the easier task typically shows higher task-switching costs than the more difficult task (Allport, Styles, & Hsieh, 1994; Monsell, Yeung, & Azuma, 2000).

## General discussion

Based on existing theories, we tested if validity sequence effects depend on feature and response repetitions. Prior research indicated that peripheral cues have a stronger validity effect following preceding valid than invalid trials (Goller & Ansorge, 2015), but the underlying principles are not well understood. As the priming account and the event-file coding explanation of inter-trial sequence effects predicted a dependence of the validity sequence effect on the presence of the same stimulus features and even the same responses in trials *n*-1 and *n*, respectively, we used two tasks with different targets. From trial to trial, these tasks were repeated or switched. As full feature or event repetitions and full changes were only possible in trial-to-trial task repetition conditions, we were able to test if the validity sequence effects were indeed restricted to trial-to-trial task repetitions. Whereas, Experiments 1 and 2 suggested that validity sequence effects can cross-task boundaries, in Experiment 3, the validity sequence effect was only clearly present if both tasks and responses repeated from one trial to the next. This is evidence for an event-file coding explanation, and though response-repetition independent validity sequence effects in the error rates of Experiment 3 showed that even in this experiment not all results could be explained by priming or event-file coding, together the results of all three experiments make clear that the exact task context could determine if validity sequence effects do or do not depend on trial-to-trial similarity. To note, only in Experiment 3, task-specific cues were used so that the cues signaled which alternative task came up. In contrast, in Experiment 1, across-task validity-sequence effects were found with cues that were shared across tasks, and in Experiment 2, with cues that were more associated with one task than with the other task but that were not discriminating between alternative tasks, cross-task validity sequence effects were partly independent of trial-to-trial task and response repetitions. Together, these observations are in line with task-file updating theory (Schumacher & Hazeltine, 2016), according to which task features that differ between tasks, so as to signal alternative tasks, are incorporated into different task sets and, as a consequence, can prevent sequential modulations during trial-to-trial changes of these task-discriminating features (Hazeltine et al., 2011). These conclusions are interesting as they suggest that task contexts have the power to elicit feature-based and response-based trial-to-trial modulations that can replace other forms of sequential modulations that are unrelated to repetition priming, such as sequence effects based on the expectancy of helpful stimuli (Gratton et al. 1992) or on the suppression of harmful stimuli (Botvinick et al., 2001). Of course, evidence for one principle—in Experiment 3, for inter-trial priming or for event-file coding—is not necessarily evidence against these alternative accounts. For example, it is possible to assume that some degree of stimulus, response, or conflict similarity between two successive trials is necessary for either more suppression of irrelevant stimuli (or features) following *n*-1 incongruent than *n*-1 congruent trials or for more expectancy of helpful accessory stimuli (or features) following *n*-1 congruent than *n*-1 incongruent trials.

In addition, what is uncertain after our Experiment 3 is whether it is necessary that one of the cue features signaled the tasks and, if so, which of the cue features was responsible for setting strong boundaries around the tasks, and thus containing the validity sequence effect within the tasks. The reason for this is that the tasks in Experiment 3 differed by more than cue characteristics. For example, the responses and the target axes also differed between tasks. Although past research makes it unlikely that validity sequence effects depend on trial-to-trial position repetitions (see Experiment 2 of Goller & Ansorge, 2015), it is possible that validity sequence effects that were independent of trial-to-trial feature and response repetitions required that alternative tasks were at least characterized by the usage of the same set of responses or the same set of positions in both of these tasks. The use of the same sets of positions and responses could have invited the joint representation of the two tasks into one representation. Such influences would also be independent of the cue-to-task associations.

In any case, the stronger independence of the validity sequence effect from trial-to-trial repetitions of task and response in Experiments 1 and 2 prevents us from endorsing feature priming and event-file coding as the only logically possible explanations. By implication, alternative accounts based on the participants’ expectancies of helpful cues following a preceding valid trial (Gratton et al., 1992) or the participants’ suppression of harmful cues following a preceding invalid trial (Botvinick et al., 2001) remain as possible candidates for explaining validity sequence effects. Yet, as was clear from lacking consistent interactions between validity sequence effects on the one hand and sequential modulations of SR congruence effects on the other hand, even these alternative principles of conflict reduction or expectancy-based modulation are to some extent dimension-specific (cf. Egner, 2008).

## Conclusion

The present study showed that validity sequence effects can be of different types, depending on the type of context provided by the tasks. On the one hand, where cues are the same across different tasks and the use of the same sets of stimulus locations and responses fostered some degree of joint representation of different tasks, validity sequence effects were found that were more independent of the trial-to-trial repetition of the exact stimulus features and responses. Such validity sequence effects could be due to conflict monitoring (Botvinick et al., 2001) or they could be due to the expectations of the participants that are based on whether or not the last cue was helpful for finding the target (Gratton et al., 1992). On the other hand, once the tasks are clearly discriminated by the cues but also by their stimulus positions and by the set of the responses that were used, validity sequence effects depended on trial-to-trial feature and response repetitions. This is evidence for the priming explanation (Mayr et al., 2003) and for the event-file coding account (Hommel et al. 2004) of the validity sequence effect. Obviously, it thus depends on the exact details of the task context which kind of process accounts for validity-sequence effects, a finding in line with task-file updating accounts (Schumacher & Hazeltine, 2016).
